# The HDAC Inhibitor LBH589 Induces ERK-Dependent Prometaphase Arrest in Prostate Cancer via HDAC6 Inactivation and Down-Regulation

**DOI:** 10.1371/journal.pone.0073401

**Published:** 2013-09-04

**Authors:** Mei-Jen Chuang, Sheng-Tang Wu, Shou-Hung Tang, Xiang-Me Lai, Hsiao-Chu Lai, Kai-Hsiang Hsu, Kuang-Hui Sun, Guang-Huan Sun, Sun-Yran Chang, Dah-Shyong Yu, Pei-Wen Hsiao, Shih-Ming Huang, Tai-Lung Cha

**Affiliations:** 1 Division of Urology, Department of Surgery, Tri-Service General Hospital, National Defense Medical Center, Taipei, Taiwan, ROC; 2 Agricultural Biotechnology Research Center, Academia Sinica, Taipei, Taiwan, ROC; 3 Department of Biotechnology and Laboratory Science in Medicine, National Yang-Ming University, Taipei, Taiwan, ROC; 4 Department of Biochemistry, National Defense Medical Center, Taipei, Taiwan, ROC; 5 Taipei City Hospital, Taipei, Taiwan, ROC; 6 Department of Microbiology and Immunology, National Defense Medical Center, Taipei, Taiwan, ROC; 7 Department of Biology and Anatomy, National Defense Medical Center, Taipei, Taiwan, ROC; Innsbruck Medical University, Austria

## Abstract

Histone deacetylase inhibitors (HDACIs) have potent anti-cancer activity in a variety of cancer models. Understanding the molecular mechanisms involved in the therapeutic responsiveness of HDACI is needed before its clinical application. This study aimed to determine if a potent HDACI, LBH589 (Panobinostat), had differential therapeutic responsiveness towards LNCaP and PC-3 prostate cancer (PCa) cells. The former showed prometaphase arrest with subsequent apoptosis upon LBH589 treatment, while the latter was less sensitive and had late G2 arrest. The LBH589 treatment down-regulated HDAC6 and sustained ERK activation, and contributed to prometaphase arrest. Mechanistically, LBH589 inhibited HDAC6 activity, caused its dissociation from protein phosphatase PP1α, and increased 14-3-3ζ acetylation. Acetylated 14-3-3ζ released its mask effect on serine 259 of c-Raf and serine 216 of Cdc25C subsequent to de-phosphorylation by PP1α, which contributed to ERK activation. Enhanced ERK activity by LBH589 further down-regulated HDAC6 protein levels and sustained ERK activation by free-forward regulation. The sustained Cdc25C and ERK activation resulted in early M-phase (prometaphase) arrest and subsequent apoptosis in the most sensitive LNCaP cells but not in PC-3 cells. This study provides pre-clinical evidence that HDAC6 may serve as a sensitive therapeutic target in the treatment of prostate cancer with HDACI LBH589 for clinical translation. This study also posits a novel mechanism of HDAC6 participation in regulating the c-Raf-PP1-ERK signaling pathway and contributing to M phase cell-cycle transition.

## Introduction

The rapid development of HDAC inhibitors (HDACI) as cancer therapeutics has been fervently applied in more than 80 clinical trials [1]. Understanding the detailed molecular mechanisms of how HDACI mediates anti-cancer activity is necessary in order to successfully facilitate its clinical translation. It suppresses cancer cell survival through various mechanisms, including blocking angiogenesis, inhibiting intracellular stress response pathways, increasing the generation of reactive oxygen species, and influencing endoplasmic reticulum stress response due to impaired handling of mis-folded proteins [2–4]. Among these anti-cancer activities, HDACI-mediated G1 cell cycle arrest causes an increase in expression of the tumor suppressor gene p21 in a transcription-dependent manner [5]. It also has been shown that HDACI induces G2/M cell-cycle arrest through a transcription-independent pathway via down-regulation of Aurora A and B kinases [6–8]. In addition, HDACI triggers a G2 phase checkpoint response in normal human cells and that this checkpoint response is defective in a range of tumor cells [9]. Therefore, targeting the inhibition of G2/M cell cycle progression is a more beneficial strategy to specifically suppress the growth of cancer cells without inhibiting normal cells.

Induction of mitosis of the cell cycle is tightly regulated by coordinated activation and inactivation of multiple protein kinases and phosphatases. At the start of mitosis, Cdc25C (a dual phosphatase) activation is a critical step for the activation of the Cdc2/cyclin B complex. The inhibitory residue of Ser216 on Cdc25C must be dephosphorylated by PP1 in order to activate Cdc25C [10,11]. The full activity of Cdc25C is regulated by ERK upon mitogen stimulation during G2/M transition in mammalian cells [12]. ERK belongs to the MAPK cascade and its activity is controlled by upstream Raf/MEK signaling. In quiescent cells, 14-3-3 binds to c-Raf via S259 and S621 phosphorylation and maintains c-Raf inactivation [13,14]. When cells enter into mitosis, the PP1 or PP2A mediated dephosphorylation of S259 is a prerequisite for the phosphorylation of S338 on c-Raf and further triggers c-Raf and ERK activation [15,16].

Notably, PP1 and ERK activities are essential for Cdc25C activation at the start of mitosis, followed by decreasing ERK activity during M phase transition [12,17]. PP1 can directly bind to the catalytic subunit of the C-terminal of HDAC6. The interaction of HDAC6 and PP1 can be disrupted by inhibiting the activity of either HDAC6 or PP1 [18]. HDAC6 is the major deacetylase that is responsible for deacetylation of α-tubulin and heat shock protein 90 (Hsp90) [19]. However, whether HDAC6 contributes to the regulation of G2/M cell cycle transition remains unclear.

LBH589 (Panobinostat), a HDACI, exhibits at least a ten-fold more potent inhibitory activity against all Class I, II, and IV HDACs compared to SAHA (vorinostat) [20]. LBH589 possesses potent growth inhibition effects on various types of cancer cells, but with varying therapeutic efficacy, which may represent potential resistance of certain cancer types and hinder the clinical translation of LBH589. Although LBH589 induces G2/M cell cycle arrest through the degradation of both Aurora A and B kinases [6,7], the detailed molecular mechanisms involved as well as the potential HDACs targeted by LBH589 remains undetermined.

The present study characterized two distinct types of G2/M cell cycle arrest mediated by LBH589 in prostate cancer cells. LBH589 not only inhibited HDAC6 and enhanced 14-3-3ζ acetylation, but also depleted HDAC6 to trigger the dissociation of PP1α from HDAC6. LBH589 subsequently interfered in the regulation of the c-Raf-ERK signaling pathway, contributing to M phase cell cycle transition. In conclusion, this study suggests that HDAC6 may be a sensitive therapeutic target in the treatment of prostate cancer using LBH589 for clinical translation in future.

## Results

### LBH589 induced G2/M cell-cycle arrest and growth inhibition in prostate cancer cells through distinct mechanisms

**Figure 1:**
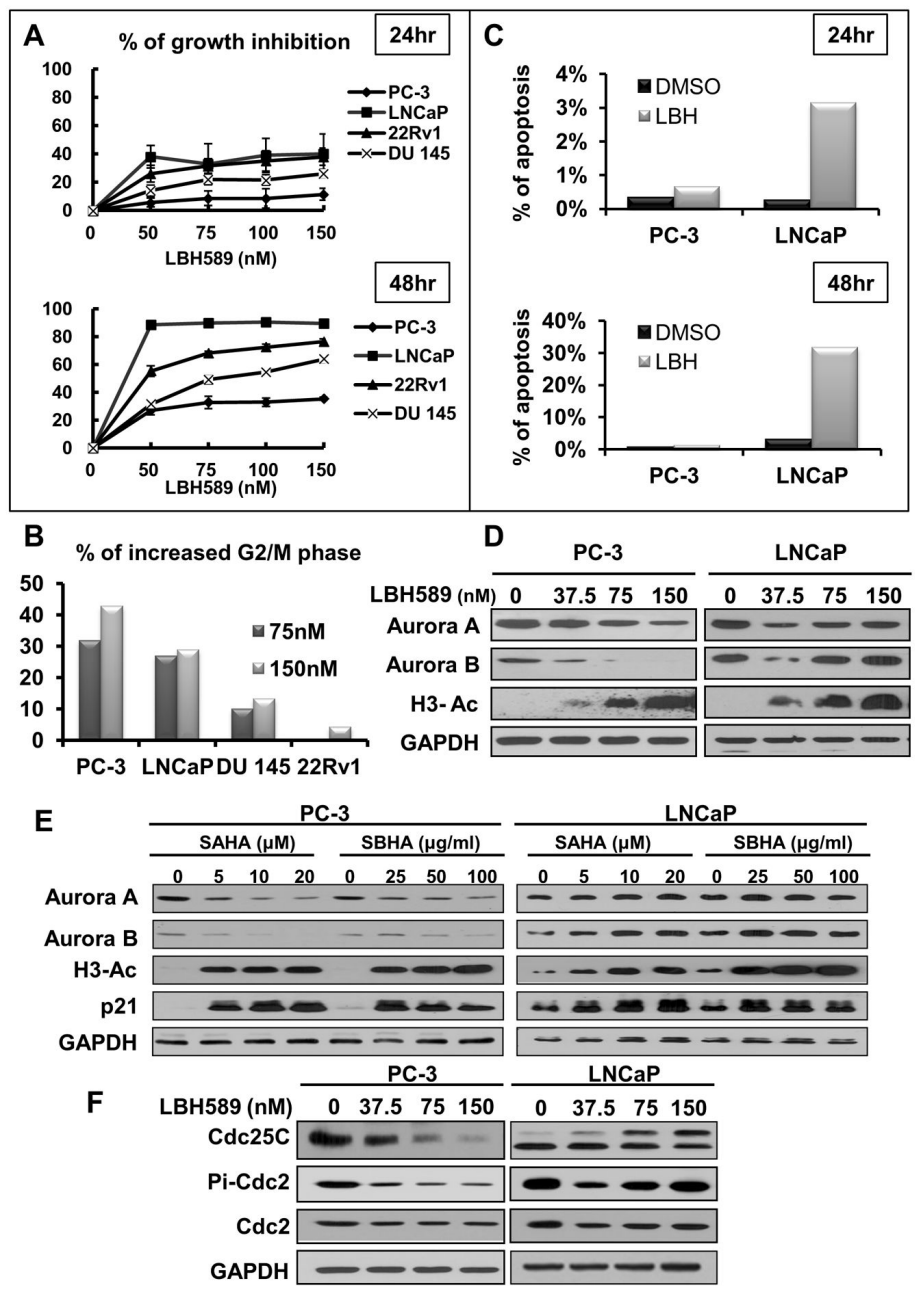
HDACI induced G2/M phase cell cycle and growth inhibition in prostate cancer cells via distinct mechanisms. (**A**) LBH589 had a dose-dependent effect on growth inhibition in prostate cancer cell lines. The cell lines were treated with 50, 75, 100, and 150 nM of LBH589. Cell survival was measured using MTT assay as described in the Materials and Methods section. Graphs compared the percentage of growth inhibition to vehicle control at 24 (upper) and 48 (lower) hours. (**B**) LBH589 induced G2/M cell cycle arrest. All cell lines were treated with LBH589 or vehicle control for 24 h. The cell cycles were analyzed by PI-staining and flow cytometry according to DNA content. The increased percentages of G2/M cells were compared to that of vehicle control. (**C**) LBH589 induced apoptosis in PC-3 and LNCaP cells. Both cell lines were treated with 75nM LBH589 or vehicle control for 24 (upper) and 48 (lower) h. The percentages of apoptotic cells were analyzed by counting the population of DNA fragmentation by flow cytometry. (**D**–**E**) HDACIs induced Aurora kinase degradation found in PC-3 but not in LNCaP. PC-3 and LNCaP cells were treated with different HDACIs at indicated concentrations of LBH589, SAHA and SBHA for 24 h and cell lysates were analyzed by western blotting using the indicated antibodies. (**F**) Profile of proteins regarding progression of G2/M cell cycle. The cells were treated with different dosages of LBH589 for 24 h. The lysate was analyzed by western blotting.

As an initial attempt to investigate the cytotoxic effect of LBH589, four prostate cancer cell lines, LNCaP, PC-3, DU-145 and 22Rv1, were treated with various concentrations of LBH589 and cell viability was measured by MTT assay. In general, LBH589 showed potent growth inhibition effect on each cancer cell line in a dose- and time-dependent manner. Among the cell lines, LNCaP and PC-3 were the most sensitive and resistant to LBH589 treatment, respectively (Figure 1A). Investigating the cell cycle profiles subsequent to LBH589 exposure, LBH589 treatment did not cause G2-M, but not G1, growth arrest in the four prostate cancer cell lines for 24 hours (Figure 1B). LBH589-induced G2-M growth arrest was most prominent in PC-3 and LNCaP. Although LBH589 induced a comparable degree of G2-M arrest in LNCaP and PC-3 cells, the significant difference of apoptosis between these two cell lines under prolonged LBH589 exposure (Figure 1C) suggested that the underlying mechanisms involved might be different.

A previous study has shown LBH589-mediated G2/M cell-cycle arrest via down-regulation of Aurora A and B kinase in renal cell carcinoma [6]. LBH589 treatment increased the same levels of histone H3 acetylation in both LNCaP and PC-3 cells, indicating that LBH589 was functional in both prostate cancer cell lines. However, down-regulation of Aurora A and B kinases by LBH589 was only observed in PC-3 cells but not in LNCaP cells (Figure 1D). In order to investigate whether other hydroxamic acid derivatives had similar effects on LNCaP and PC-3 cells, two other HDACIs, SAHA and SBHA, were tested and showed that the down-regulation of Aurora A and B kinases only occurred in PC-3 cells but not in LNCaP (Figure 1E). To further address the discrepancy between LNCaP and PC-3 cells under LBH589 treatment, two G2-M transition molecules Cdc2 and Cdc25C were compared. LBH589 decreased Cdc25C and phosphorylated Cdc2 levels in PC-3 cells but not in LNCaP cells. LBH589 also induced a band shift pattern of Cdc25C in LNCaP cells in a dose- and time-dependent manner (Figure 1F).

### LBH589 induced ERK activation and Cdc25C hyper-phosphorylation

**Figure 2:**
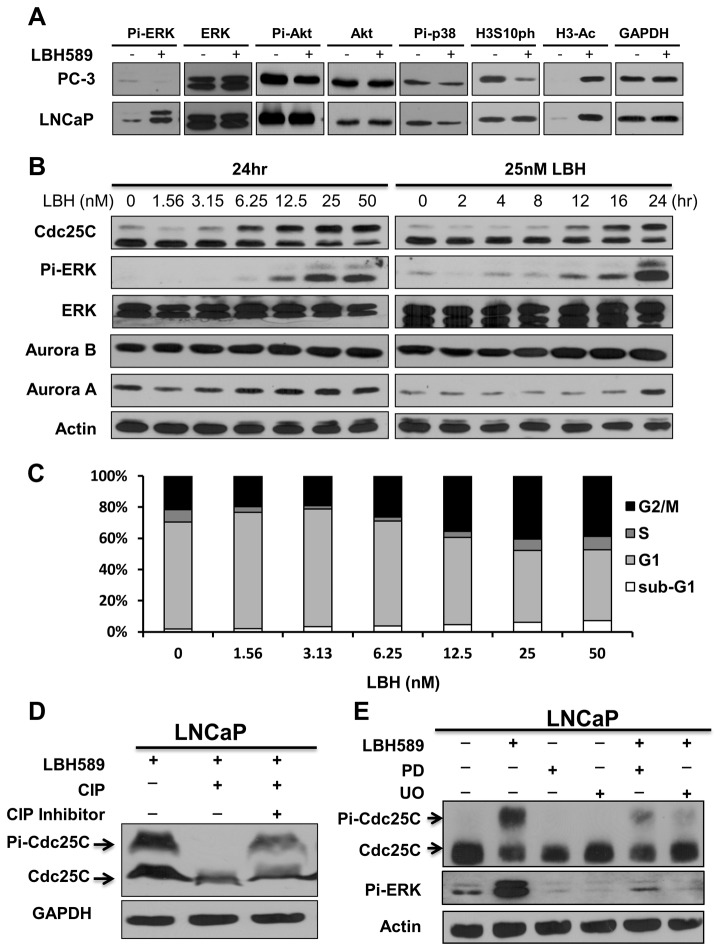
LBH589 induced ERK activation in LNCaP but not PC-3 cells. (**A**) LBH589 induced ERK activity is selectively activated in LNCaP cell. PC-3 and LNCaP cells were treated with 75nM LBH589 or vehicle control for 24 h. The levels of indicated proteins were immuno-blotted against individual antibodies. GAPDH was an internal loading control. (**B**) Dose- and time-dependent correlations of LBH589-mediated ERK activation and Cdc25C hyper-phosphorylation. LNCaP was exposed to various concentration of LBH589 for 24 h (left) or 25 nM LBH589 at different times (right). (**C**) Dose-dependent correlation of LBH589-induced G2/M arrest. LNCaP was treated with various concentrations of LBH589 for 24 h. Cell cycles were analyzed by flow cytometry. (**D**) Cdc25C hyper-phosphorylation was confirmed by dephosphorylation assay. LNCaP was treated with 50nM LBH589 for 24 hours. The lysates were incubated with phosphatase (CIP) or combined with phosphatase inhibitor. The hyper-phosphorylated and dephosphorylated Cdc25C were analyzed by immuno-blotting with Cdc25C antibody. (**E**) Cdc25C hyper-phosphorylation was suppressed by MEK inhibitors. LNCaP were treated with MEK inhibitors, PD (100 µM PD98059) or UO (20 µM UO126) for 30 minutes. LBH589 was then added to the culture after 24 h. ERK activity and Cdc25C hyper-phosphorylation were analyzed by Pi-ERK and Cdc25C antibodies in western blotting.

The molecular mechanisms involved in the distinct LBH589-mediated effects between the two PCa cells were investigated. The signaling pathways involved in cell proliferation and survival, including Akt and p38, showed no significant difference between LNCaP and PC-3 PCa cells (Figure 2A). Interestingly, LBH589 treatment resulted in increased ERK phosphorylation only in LNCaP cells, not in PC-3 cells. Down-regulation of the histone H3 serine 10 phosphorylation (as an M-phase marker) occurred in PC-3 cells, but not in LNCaP cells (Figure 2A).

Moreover, LBH589 treatment resulted in increased ERK phosphorylation and band shift of Cdc25C in a dose- and time-dependent manner in LNCaP cells (Fig. 2B), which correlated with the increasing population of G2/M cell phase (Fig. 2C). To investigate whether the band shift of Cdc25C represented hyper-phosphorylation under LBH589 treatment, the LBH589 treated LNCaP lysate was treated with CIP (phosphatase) alone, or combined treated with a phosphatase inhibitor. The band shift of Cdc25C induced by LBH589 was removed by CIP, but was restored by the phosphatase inhibitor (Figure 2D), indicating Cdc25C hyper-phosphorylation.

Further investigating the correlation between ERK activation and Cdc25C hyper-phosphorylation of cells under LBH589 treatment, the Cdc25C hyper-phosphorylation induced by LBH589 was blocked by MEK inhibitors U0126 and PD98059 in LNCaP cells. This indicated that Cdc25C hyper-phosphorylation was a downstream event of LBH589-induced ERK activation (Figure 2E).

### LBH589 induced G2 or M phase arrest and ERK activation and contributed to prometaphase cell-cycle arrest

**Figure 3:**
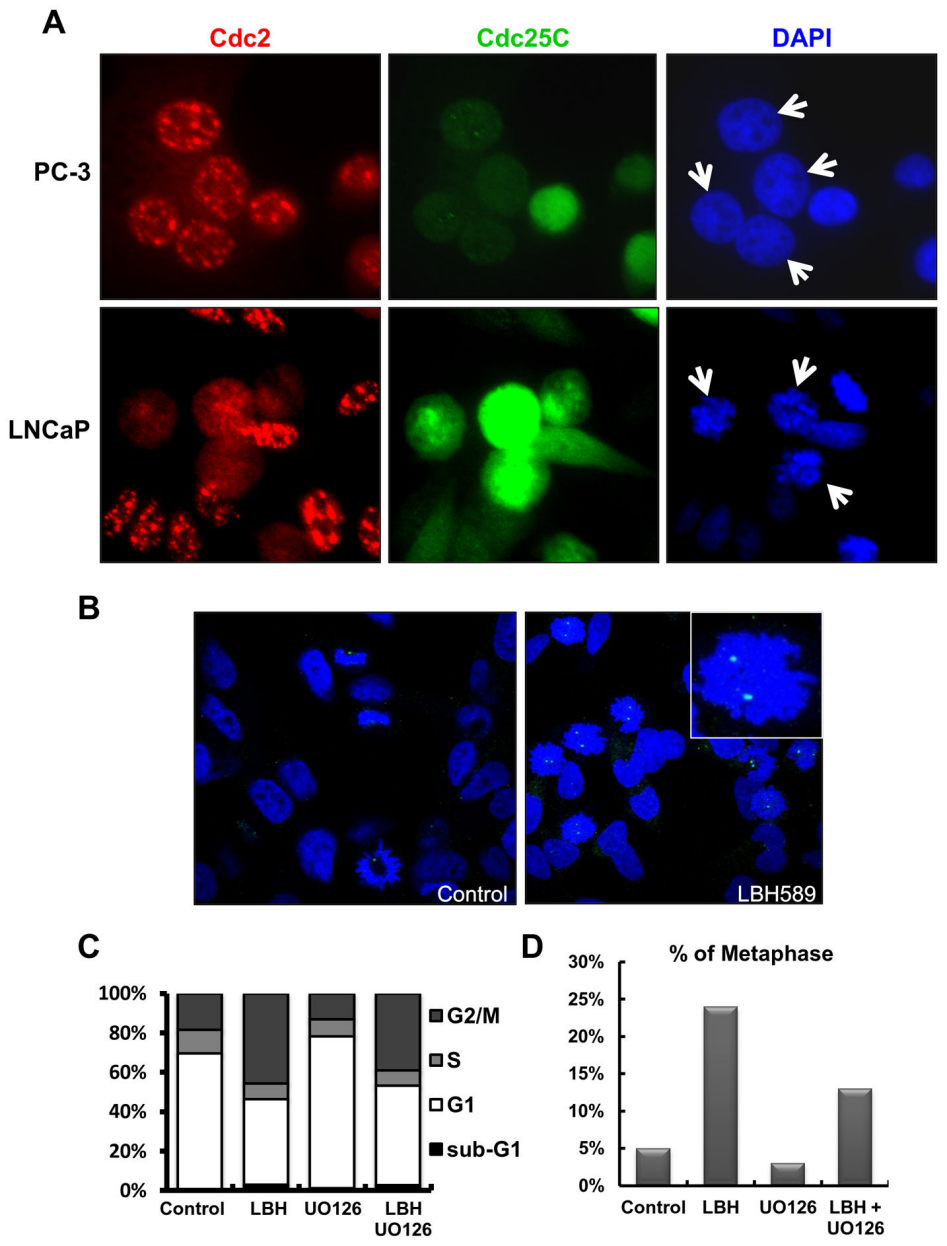
LBH589 induced prometaphase arrest in LNCaP but not in PC-3 cells. (**A**–**B**) Immuno-staining of LNCaP and PC-3. Both cells were treated 75 nM LBH589 for 24 h. Immuno-staining (**A**) with Cdc2 and Cdc25C antibodies and (**B**) of γ-tubulin (Green) and DAPI (Blue) in vehicle control (left) and LBH589 treatment (right) in LNCaP. (**C**–**D**) MEK inhibitor attenuated LBH589-induced prometaphase arrest in LNCaP. The cells were pre-treated 30 with UO126 for 30 minutes and sequentially treated with LBH589 for 24 h. (**C**) The cell cycles were analyzed by PI-staining and flow cytometry. (**D**) The metaphase cells were counted by DAPI staining presented as condensate chromatin.

By immuno-fluorescence, the distribution patterns of Cdc2 and Cdc25C between LNCaP and PC-3 cells were further investigated. LBH589 treatment resulted in a loss of immuno-fluorescence intensity of Cdc25C in PC-3 cells but not in LNCaP cells. Cell morphology and DAPI nuclear staining representing LBH589-treated PC-3 and LNCaP cells were arrested in late G2 and early M phase, respectively (Figure 3A, green, arrows). To determine if LBH589 induced cell arrested in the G2 or M phase, the effect of LBH589 on centrosome separation, the morphologic hallmarks of early mitosis, were investigated by immuno-staining with γ-tubulin antibodies. The separation of two centrosomes was observed only in LBH589-treated LNCaP cells (Figure 3B), suggesting that upon LBH589 treatment, PC-3 cells were arrested in the late G2 phase and LNCaP cells were arrested in the early M phase (most likely prometaphase).

To investigate the involvement of ERK activation, LNCaP cells were treated with the MEK inhibitor, U0126, to inhibit ERK activity in the presence of LBH589. The LBH589-mediated G2/M arrest was attenuated by ERK inhibition in cells treated with UO126 (Figure 3C). Furthermore, ERK inhibition reduced LBH589-mediated prometaphase cells from 24% to 13% (Figure 3D), suggesting that ERK activation played an important role in LBH589-mediated prometaphase arrest in LNCaP cells.

### LBH589 down-regulated HDAC6 and sustained ERK activation

**Figure 4:**
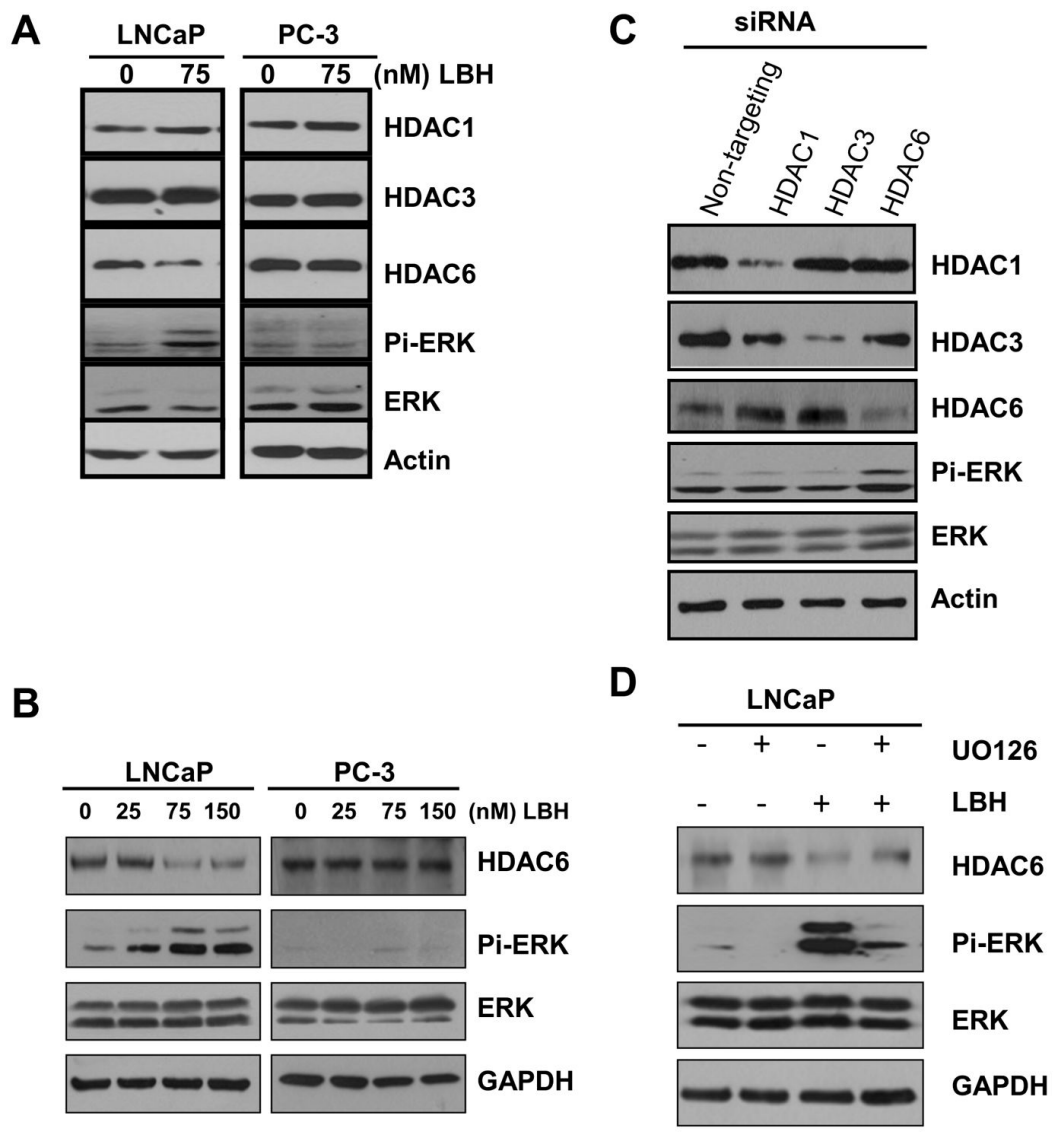
HDAC6 down-regulation correlated with ERK activation in LBH589 treatment. (**A**) Screening of HDACs involved in ERK activation. The immuno-blottings of LBH589 treated cell lysates were performed with indicated antibodies. (**B**) LBH589 induced the down-regulation of HDAC6, which correlated with ERK activation in LNCaP but not in PC-3. (**C**) Knockdown of HDAC6 increased in ERK activity. 293T was transfected with siRNA of HDAC1, 3, 6, or non-targeting for 72 hours. The lysates were analyzed by immuno-blotting. (**D**) ERK activity was involved in LBH589-mediated HDAC6 down-regulation. Cell lysates from cells treated with LBH589 or combined with UO126 pre-treatment were immuno-blotted.

The effects of LBH589 on HDAC1, HDAC3 and HDAC6 were checked. LBH589 down-regulated HDAC6 in LNCaP but not in PC-3 cells (Figure 4A). In addition, the LBH589-mediated down-regulation of HDAC6 correlated with ERK activation in dose-dependent manner but only found in LNCaP cells (Figure 4B). In order to identify which HDAC was responsible for ERK activation, the three proteins were knocked down with siRNA to examine the ERK phosphorylation status in 293T cells, since 293T cells had high transfection efficiency and LBH589 treatment also resulted in prometaphase arrest and ERK activation of 293T cells (Figures S1, S2, S3). Knock-down of HDAC6, but not HDAC1 or HDAC3, by siRNA significantly induced ERK phosphorylation in 293T cells (Figure 4C).

The LNCaP cells were then treated with LBH589 and the MEK inhibitor, U0126. Inhibition of LBH589-induced ERK activation restored HDAC6 protein levels (Figure 4D). Furthermore, ERK was over-expressed in 293T cells, which resulted in the down-regulation of HDAC6 protein levels compared to EGFP-vector alone (data not shown). These demonstrated that inhibition of HDAC6 activity by LBH589 induced ERK activation, which subsequently led to the down-regulation of HDAC6 protein levels. A reciprocal regulation might therefore exist between HDAC6 and the ERK signaling pathway.

### LBH589 induced ERK activation through the PP1/c-Raf pathway

**Figure 5:**
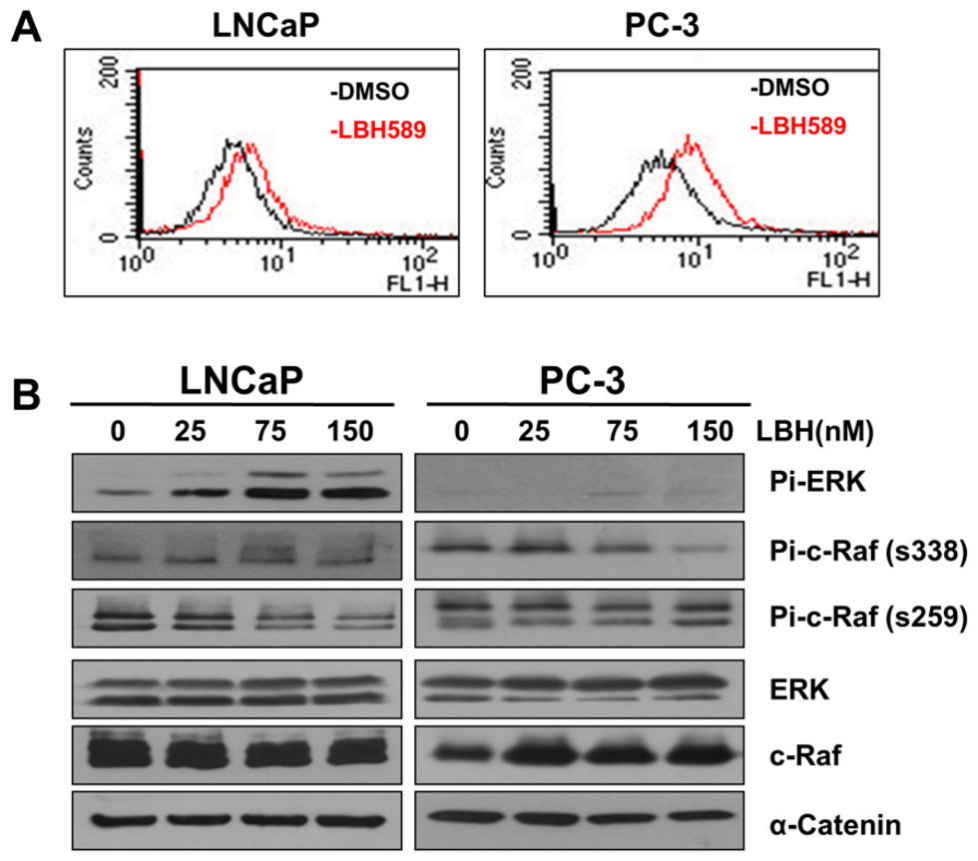
LBH589 induced ERK activation by modulating c-Raf activity. (**A**) Analysis of ROS production. The indicated cells were treated with 75 nM LBH589 for 24 h. ROS was measured as described in the Materials and Methods section. (**B**) The pattern of c-Raf signaling pathway on LBH589 treatment. The cells were treated with LBH589 for 24 h and the lysates were immuno-blotted with the indicated antibodies. α-catenin was a loading control.

A previous study showed that HDACI might increase intracellular ROS levels and activate the Ras-Raf signaling pathway [21]. The ROS levels of LNCaP and PC-3 cells under LBH589 treatment were then investigated. LBH589 treatment induced prometaphase arrest but did not significantly increase intracellular ROS levels in LNCaP, but induced a certain level in PC-3 cells (Figure 5A). LBH589 treatment also decreased the serine 259 phosphorylation (inhibitory signal) and increased the serine 338 phosphorylation (activating signal) of c-Raf, which correlated with ERK activation in LNCaP but not in PC-3 cells (Figure 5B). These suggested that the LBH589-induced ERK activation might be mediated via c-Raf activation.

### LBH589 switched PP1 interaction with 14-3-3ζ and HDAC6

**Figure 6:**
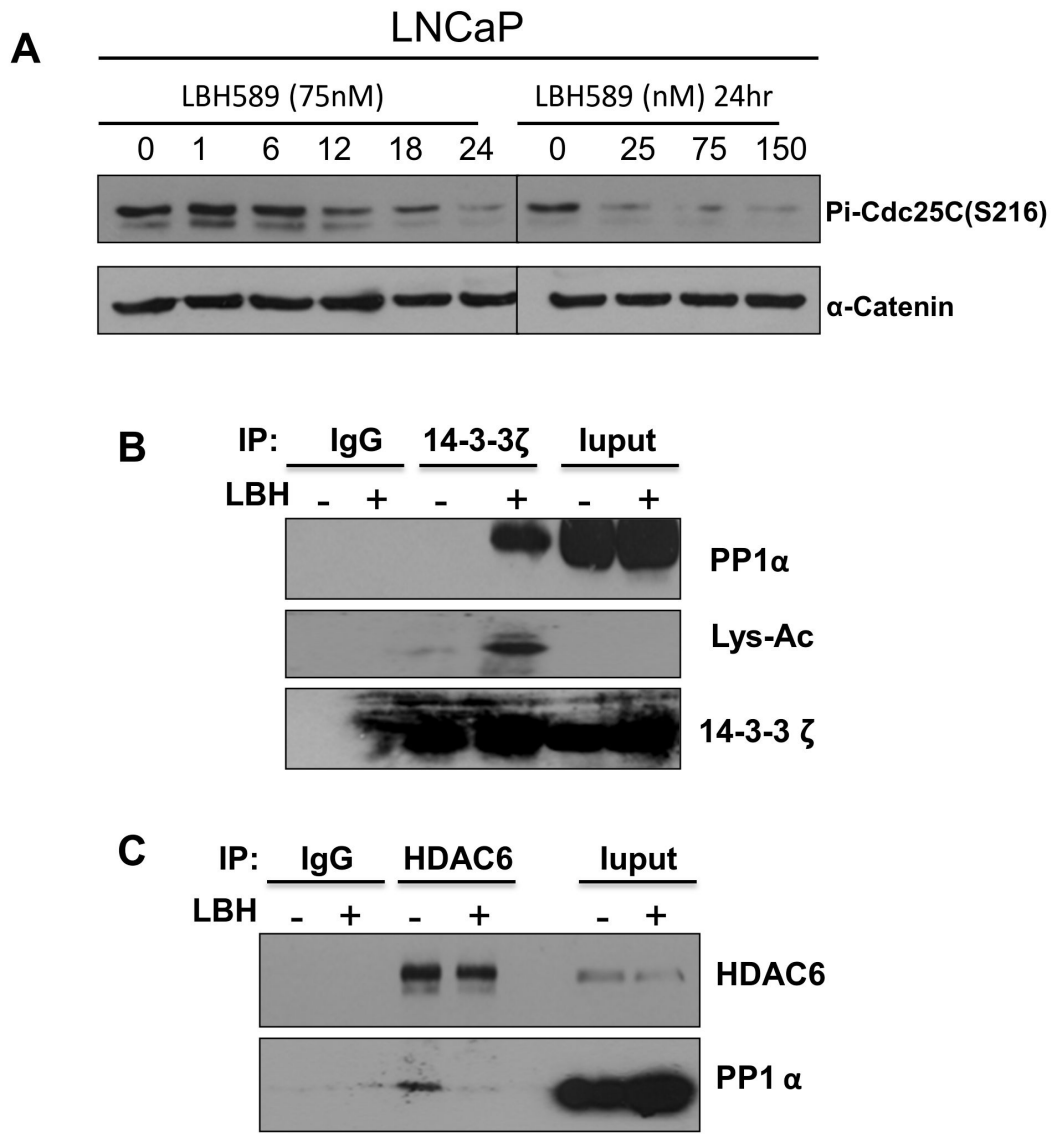
LBH589 switched the PP1 interacting partner for ERK activation. (**A**) LBH589 induced Cdc25C-S216 dephosphorylation in a dose- and time-dependent manner. LNCaP was exposed to 75 nM LBH589 for various times or at indicated LBH589 concentrations for 24 h. Phosphorylation of Cdc25C-S216 was performed by immuno-blotting with Pi-Cdc25C-S216 antibody. (**B**–**C**) LBH589 treatment switched the PP1α interacting partner. The immuno-precipitations were conducted with anti-HDAC6 or anti-14-3-3ζ. The precipitated samples were analyzed by immuno-blotting using antibody against PP1α, 14-3-3ζ, Lys-Ac (Pan-acetylated lysine). IgG was a nonspecific pull-down control.

Over-expression of PP1α, but not PP2A, enhanced the dephosphorylation of c-Raf Ser259 and phosphorylation of Ser338, and then further activated downstream ERK phosphorylation under LBH589 treatment in 293T cells (data not shown). These results suggested that LBH589 might trigger PP1α to activate c-Raf by dephosphorylating Ser259 and subsequently activating ERK. Because both phosphates of Cdc25C-Ser216 and c-Raf-Ser259 were substrates for PP1, the effect of LBH589 on Ser216 phosphorylation of Cdc25C was further examined. LBH589 treatment reduced the Ser216 phosphorylation of Cdc25C in a dose- and time-dependent manner in LNCaP cells (Figure 6A).

14-3-3 was a chaperon protein to maintain the phosphorylation status of S216 of Cdc25C and S259 of c-Raf by protecting them from dephosphorylation by PP1 [10,22]. The interaction between 14-3-3, PP1α, and HDAC6 was checked using 14-3-3**ζ**, PP1α and HDAC6 antibodies in the co-immuno-precipitation analysis. LBH589 treatment increased 14-3-3**ζ** acetylation and its interaction with PP1α (Figure 6B). In contrast, HDAC6 decreased its interaction with PP1α after LBH589 treatment (Figure 6C). These indicated that HDAC6 might be responsible for 14-3-3**ζ** deacetylation to protect its client proteins c-Raf and Cdc25C from dephosphorylation by PP1α.

## Discussion

A previous study reveals that LBH589 treatment causes G2/M cell-cycle arrest via degradation of Aurora kinases in renal cell carcinoma (RCC) [6]. Similarly, the present study further clarifies that LBH589 induces a late G2 phase arrest through the down-regulation of Aurora kinases in PC-3 prostate cancer cells with relative resistance to LBH589 treatment. HDACIs trigger a G2 checkpoint in normal cells that is usually defective in cancer cells, which provides an explanation why normal cells are usually more resistant to HDACI treatment [9]. The underlying mechanisms involved in LBH589 mediated G2 phase arrest between normal cells and prostate cancer cells may be different. However, results here imply that the LBH589-induced G2 arrest may be a phenotype of cancer cells that are more tolerant to LBH589 treatment.

In contrast to LBH589-mediated G2 phase arrest of PC-3 cells, LBH589 induces prometaphase arrest subsequent to profound apoptosis of LNCaP cells. Although the genetic backgrounds of prostate cancer cell lines are different, LBH589 treatment induces ERK activation and HDAC6 down-regulation in 22Rv1 and DU 145 PCa cell lines other than LNCaP cells (Figure S4). However, the prominent prometaphase arrest only occurs in PCa cells with low baseline levels of ERK phosphorylation, which can be sustainably induced by LBH589. LBH589 transiently induces ERK activation after one hour of treatment, which is quickly eliminated within 6 hours after LBH589 treatment in PC-3 (data not shown).

Inhibition of HDAC6 activity by LBH589 only induces transient ERK activation and no HDAC6 protein level change is noted in PC-3 cells. This phenomenon is similar to transient ERK activation induced by mitogens, such as EGF in PC-3 cells. This study shows that the inhibition of HDAC6 by LBH589 directly up-regulates ERK activation through the c-Raf/PP1 pathway, which is similar to mitogen stimulation but with a totally opposite biological outcome of cell-cycle arrest instead of cell proliferation.

ERK activation, which responds to a variety of extracellular stimuli such as mitogens or stresses, results in dramatically different biological consequences including proliferation, differentiation, and cell death [23–25]. Constant ERK activation can induce cell death when cells were under stress [26]. LBH589 induces sustained ERK activation through a free forward regulation between HDAC6 and ERK in cancer cells, which not only induces prometaphase arrest but also triggers apoptosis. There is no detectible expression change of HDAC6 mRNA in LNCaP cells after LBH589 treatment (data not shown). The proteasome inhibitor restores HDAC6 protein levels after LBH589 treatment, suggesting that the depletion of HDAC6 proteins may depend on a proteasome-mediated degradation pathway (data not shown). However, why LBH589 selectively down-regulating HDAC6 proteins and Aurora kinases A and B in certain specific cancer cell lines remains unclear.

HDAC6 belongs to the Class IIa subtype of HDAC families. It shuttles between the nucleus and cytoplasm to achieve its biological functions. HDAC6 is the major deacetylase that is responsible for deacetylation of α-tubulin and HSP90 to modulate microtubulin-dependent transportation, recruit mis-folded proteins, and transport to aggresomes for degradation [27–29]. Aside from participating in many normal cellular functions, HDAC6 may be required for efficient oncogenic transformation and may be involved in the cascade of the transforming growth factor β1 (TGF-β1)-induced epithelial-mesenchymal transition [30,31]. These indicate the important roles of HDAC6 in oncogenic processes.

It has been reported that acute myeloid leukemia cells lacking in HDAC6 are highly resistant to hydroxamate group HDACIs [32]. Thus, HDAC6 may serve as a pivotal therapeutic target for HDACIs in cancer treatment. Since LBH589 is a potent hydroxamate group HDACI with strong inhibitory effect on HDAC6, LBH589 has the advantage of clinical application in the treatment of cancers with HDAC6 expression. Although the enzyme activity of HDAC6 can be inhibited by LBH589 in both LNCaP and PC-3 PCa cells, LBH589 selectively depletes either HDAC6 or Aurora kinases in LNCaP and PC-3 PCa cells with distinct biological outcomes, respectively. This study raises the important question of why LBH589 selectively depletes either HDAC6 or Aurora kinases through a proteasome degradation pathway in different PCa cells. Understanding the molecular mechanisms behind this discrepancy in the therapeutic response of LBH589 on different PCa cells can provide more insights for the clinical application of LBH589.

The results here prove that LBH589 induces ERK activation by inhibiting HDAC6 activity in certain cells. ERK activation is controlled by the upstream Ras/Raf/MEK pathway [33]. Dephosphorylation of S259 of c-Raf by two phosphatases, PP1 or PP2A, results in c-Raf release from 14-3-3 and allows for the reactivation of c-Raf, which in turn triggers ERK activity [34]. HDAC1, 6, and 10 have been reported to form a complex with PP1, respectively. HDACIs selectively disrupt the HDAC-PP1 complex and increase the association of PP1 and Akt, which contributes to the anti-neoplastic activities of HDACI [35]. The present study shows that LBH589 disrupts the HDAC6/PP1α complex and promotes the interaction between PP1α and acetylated 14-3-3ζ. When PP1α is associated with 14-3-3ζ, PP1α still maintains its phosphatase activity [36]. With LBH589 switching its interacting partner, PP1α may alter its affinity or specificity to substrates. Again, an important question is raised as to whether HDACs are involved in cell cycle regulation by altering the substrates’ affinity or specificity of PP1α.

In addition to ERK activation, inhibition of HDAC6 by LBH589 also induces Cdc25C hyper-phosphorylation by removal of inhibitory phosphorylation of serine 216 of Cdc25C. LBH589-induced dephosphorylation of S216 of Cdc25C is also regulated by PP1α and 14-3-3ζ with the same mechanisms responsible for S259 dephosphorylation of c-Raf. Thus, HDAC6 not only participates in the regulation of c-Raf/PP1/ERK signaling pathway but also coordinates the ERK signaling cascade to M phase cell cycle transition.

**Figure 7:**
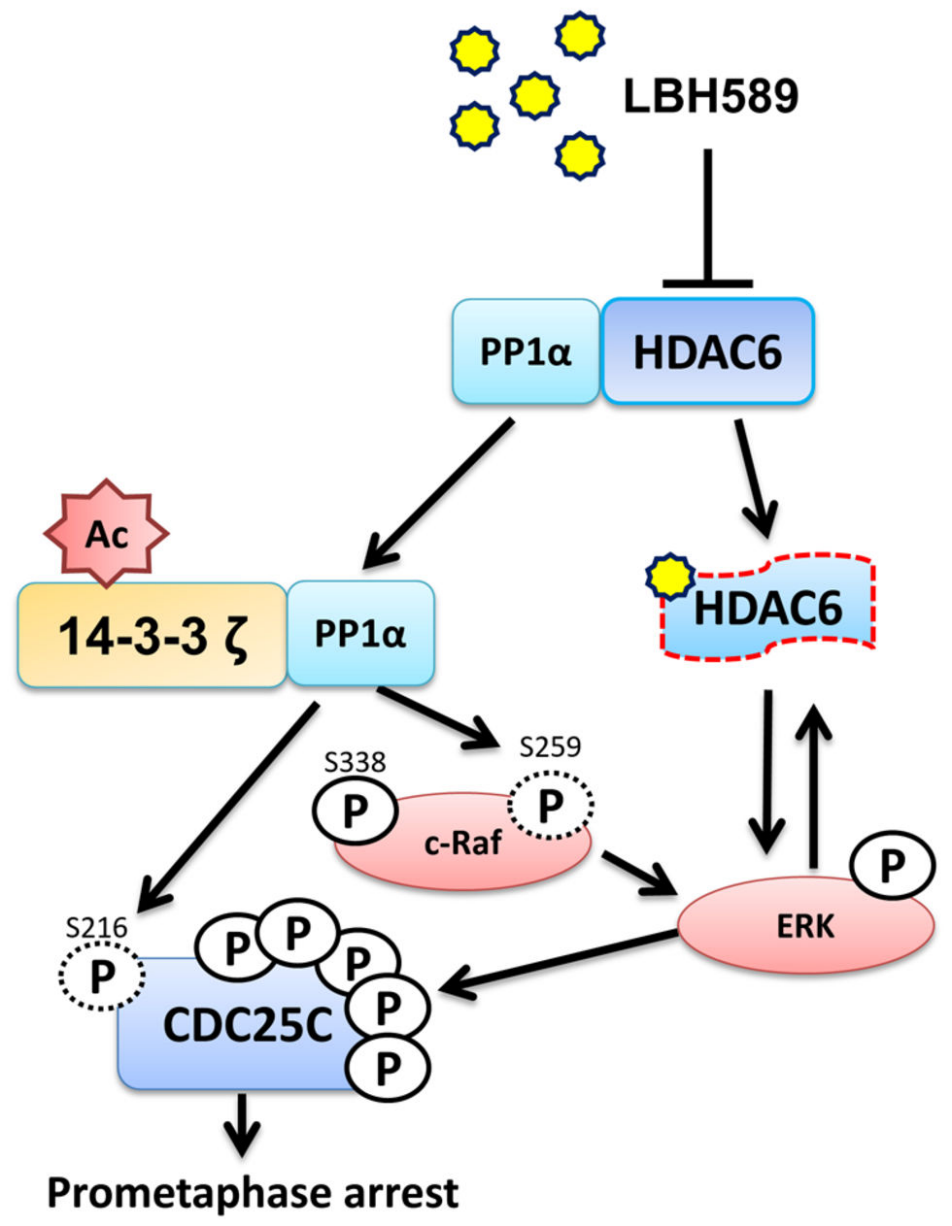
The model of LBH589-induced prometaphase arrest. LBH589 binding to HDAC6 might cause conformational change on HDAC6, leading to the dissociation of PP1α and enhancement of 14-3-3ζ acetylation. Acetylated 14-3-3ζ increased its interacting affinity with PP1α and interfered in the affinity of PP1α binding with substrate. PP1α then dephosphorylated S259 of c-Raf and S216 of Cdc25C, triggering ERK activation. Constant ERK activation due to the activation of c-Raf might destabilize HDAC6 proteins and hyper-phosphorylate CDC25C, leading to the prometaphase cell cycle arrest of LNCaP cells.

This study proposes a model to explain how LBH589 induces prometaphase arrest. When HDAC6 binds with an HDACI, such as LBH589 in this study, it may cause a conformational change in HDAC6, leading to the dissociation of PP1α and the enhancement of 14-3-3**ζ** acetylation. Acetylated 14-3-3ζ has high affinity for binding with PP1α and modulating the affinity of PP1α binding to its substrates. Further, the phosphates of c-Raf-Ser259 and Cdc25C-Ser216 are dephosphorylated by PP1α and these induce constant ERK activation. Sustain ERK activation may destabilize HDAC6 proteins and hyper-phosphorylate Cdc25C, leading to the prometaphase cell cycle arrest of LNCaP cells (Figure 7).

In conclusion, LBH589 induces sustained ERK activation through free forward regulation that inhibits HDAC6 enzyme activity, followed by the down-regulation of HDAC6 protein expression. These findings answer the question as to how ERK activation can be responsible for both cell proliferation and growth inhibition phenotypes. HDAC6 is a pivotal factor essential for the interplay between Raf/ERK signaling pathway and biological M phase cell-cycle transition, which makes it a perfect target for HDACI LBH589. This study further elucidates the LBH589-mediated late G2 and early M phase cell-cycle arrest through distinct molecular mechanisms in PC-3 and LNCaP PCa cells with different therapeutic outcomes. Therefore, the detailed mechanisms responsible for LBH589-mediated growth inhibition of prostate cancer cells are unraveled to provide new insights that may guide the design of more effective strategies that will optimize HDACI anti-cancer therapy for clinical translation.

## Materials and Methods

### Reagents

LBH589 was provided by Novartis Pharmaceuticals (East Hanover, NJ) dissolved in dimethylsulphoxide (DMSO). Suberoyl Bishydroxamic Acid (SBHA) and Suberoylanilide hydroxamic acid (SAHA) were from ATON Pharma (Tarrytown, NY). Propidium iodide (PI), 3-(4,5-dimethylthiazol-2-yl)-2,5-diphenyltetrazolium bromide (MTT), RNase A and a protease inhibitors cocktail were purchased from Sigma-Aldrich (St. Louis, MO). UO126 and PD98059 were purchased from Calbiochem.

### Cell culture

LNCaP, PC-3, and 22Rv1 were kindly provided by Dr. Pei-Wen Hsiao (Agricultural Biotechnology Research Center, Academia Sinica, Taiwan) [37]. DU 145 and 293T were purchased from Bioresource Collection and Research Center (BCRC, Taiwan). PC-3 and 293T cell lines were maintained in Dulbecco’s modified Eagle’s medium, while LNCaP and 22Rv1 were cultured in RPMI-1640. The DU 145 were maintained in modified Eagle’s medium. All media were supplemented with 10% heat-inactivated fetal bovine serum, 2 mM glutamine, 1 mM sodium pyruvate, 50 U/ml penicillin, and 50 mg/ml streptomycin. The cells were cultured at 37°C in a 5% humidified CO_2_/air atmosphere.

### MTT assays

The cells were plated in 96-well plates at a density of 3000-5000 cells per well, depending on the cell line, treated with vehicle (DMSO) or different doses of LBH589, SAHA, and SBHA using five wells per treatment. At 24, 48 and 72 h of treatment, the media were moved and 100 µl of 1mg/ml MTT was added before incubation at 37°C. After 3 h, the supernatant was removed and 100 µl of DMSO was added to dissolve the crystal. Colorimetric absorbance was determined at 560 nm in a micro-plate reader (Quant, Bio-Tek Instruments, Inc., Winooski, VT).

### Flow cytometry

The cells were plated in six-well plates and treated with LBH589 at different concentrations. After 24 hours, the cells were trypsinized, washed with PBS, fixed with 70% cold ethanol at -20^°^C for at least one h, treated with 0.5% Triton X-100 and 0.05% RNase A in PBS for 30 min, and stained with propidium iodide. The cells were analyzed using an FACSCalibur flow cytometer (BD Biosciences) to determine cell cycle distribution of DNA content.

### Western blotting

The cells were seeded in a 100 mm dish before 24-h treatment and then treated with different doses of LBH589, SAHA, and SBHA. After 24 h, the cells were lysed in an ice-cold RIPA buffer containing 50 mM Tris pH 7.4, 1% NP40, 150 mM NaCl, 40 mM NaF, 1 mM Na _3_VO_4_, 1 mM EDTA, and a protease inhibitor cocktail. Western blotting was performed as previously described [6].

### Antibodies

The antibodies included HDAC1 (#2062), c-Raf (#9422), Akt (#9272), Pi-Akt (#4058), Pi-Cdc25C (Ser216) (#9528), Pi-c-Raf (S259) (# 9421), Pi-c-Raf (S338) (#9427), and Pi-Cdc2 (Tyr15) (#9111) purchased from Cell Signaling Technology (Beverly, MA, USA); HDAC6 (sc-28386), Pi-ERK(sc-7383), ERK (sc-94), 14-3-3ζ (sc-1019), and Cdc2 (sc-54) from Santa Cruz Biotechnology (Santa Cruz, CA, USA); Aurora A (#07-648), H3-Ac (#06-599), H4-Ac (#06-598), p21 (#05-345), and PP2A (#05-421) from Millipore (Lake Placid, NY, USA); GAPDH and actin (Sigma-Aldrich); Aurora B (#1788-1), Cdc25C (#1302-1), and PP1 (#1950-1) from Epitomics (Burlingame, CA); and HDAC3 (ab32369) from Abcam (Cambridge, UK).

### Immuno-staining

The PC-3 and LNCaP cells were grown on poly-lysine coated coverslips for 48 h, and then treated with 75nM LBH589 for 24 h. The cells were fixed with 4% paraformaldehyde-PBS for 30 min, further washed with 0.1% Tween 20-PBS (PBST) twice, and permeabilized by incubation in PBS containing 0.1% Triton X-100 for 30 min. The cells were then washed twice and blocked with 5% BSA in TBST for 1 h, incubated with a primary antibody overnight at 4°C, washed with PBST, and incubated in Alexa 488 (Green)- or Alexa 594 (Red)-conjugated secondary antibody containing DAPI for 3 h. Lastly, the cells were washed using coverslips with PBST and mounted with anti-fade mounting medium.

### Immuno-precipitation (IP)

LNCaP was treated with 75 nM LBH589 or DMSO control for 12 h. The cells were washed with PBS and lysed in IP-14-3-3 buffer (20 mM Tris, pH7.4 containing 150 mM NaCl, 1 mM EGTA, 10 M EDTA and 1% Triton X-100) or IP-HDAC6 buffer (10 mM Tris, pH7.4 containing 150 mM NaCl, 0.5% NP-40 and 1 mM EDTA). Both buffers contained a protease inhibitor cocktail (Sigma). The lysates were centrifuged to remove debris and pre-cleaned with 20µl of protein A/G sepharose (GE). A total of 1 mg of lysate was used for each IP assay.

The lysates were incubated with IP antibody overnight. The IP antibody 5 µl of 14-3-3ζ (Enzo) was used with 10 µl of HDAC6 (Santa Cruz) and an equal amount of IgG antibody. The protein complex was then precipitated by protein A/G sepharose. After washing, the complexes were eluted in 30µl of 1.5X SDS sample buffer and subjected to SDS-PAGE and western blotting.

### ROS Analysis

The cells were treated with 150nM LBH589 for 24 h. The trypsinized cells were incubated with 10 µM H2DCFDA (Invitrogen) in PBS for 15 min at 37 ^°^C. After washing, ROS was measured by flow cytometry according to fluorescence intensity.

### Transfection and siRNA knockdown

293T cells were transfected with lipofectamine 2000 (Invitrogen) following protocol. The siRNA were from on TARGET plus SMART pool (Darmacon) and transfected following the instrument. siRNA HDAC1 (L-003493-00-0010), siRNA HDAC3 (L-003496-00-0010), siRNA HDAC6 (CTGCAAGGGATGGATCTGAAC), and Non-Targeting Pool siRNA (D-001810-10-20) lysates were collected at 72 h post-transfection. At 48 h post-transfection, the cells were treated with LBH589 and harvested at 72 h post-transfection.

### Dephosphorylation assay (CIP assay)

The cells were treated with 50nM LBH589 for 24 h, collected, washed with PBS, and then precipitated total protein with trichloroacetic acid (TCA). The pellet was dissolved in buffer (50mM Tris, pH7.5, 10mM MgCl_2_) and the pH was neutralized to 7.5 by appreciated 2M Tris base. The lysates were incubated with or without 10 µl of Alkaline phosphatase, Calf Intestinal (CIP) or combined with 25 mM of 2-glycerphosphate (Phosphatase inhibitor) for 3 h, and then subjected to SDS- PAGE and immuno-blotting [38].

## Supporting Information

Figure S1 **LBH589 induced ERK activation, G2/M arrest, and hyper-phosphorylation of Cdc25C in 293T.** The 293T cells were treated with 37.5, 75 and 150 nM of LBH589 for 24 h. (A) The dose- and time-dependent correlation of LBH589-mediated ERK activation and Cdc25C hyper-phosphorylation. (**B**) The cell cycles were analyzed by PI-staining and flow cytometry according to DNA content. (**C**) The dephosphorylation assay. The lysates were incubated with phosphatase or combined with phosphatase inhibitor. The hyper-phosphorylated and dephosphorylated Cdc25C were analyzed by immuno-blotting with Cdc25C antibody.
(TIF)

Figure S2 **LBH589-induced down-regulation of HDAC6 correlated with ERK activation in 293T.** (**A**) The LBH589-induced down-regulation of HDAC6 correlated with ERK activation. (**B**) LBH589 mediated HDAC6 down-regulation in a dose-dependent manner. (**C**) ERK activity was involved in the LBH589-mediated HDAC6 down-regulation, as shown by the immuno-blotting of lysates from cells treated with LBH589 or combined with UO126 pre-treatment.
(TIF)

Figure S3 **LBH589 induced ERK activation by modulating c-Raf activity.** (**A**) Analysis of ROS production. 293T cells were treated with 75 nM LBH589 for 24 h. (**B**) The pattern of c-Raf signaling pathway on LBH589 treatment. 293T cells were treated with LBH589 for 24 h and the lysates were immuno-blotted with the indicated antibodies. (**C**) LBH589 induced the dephosphorylation of Cdc25C-Ser216.
(TIF)

Figure S4 **LBH589 induced ERK activation and HDAC6 down-regulation in 22Rv1 and DU 145.** (**A**) Dynamic changes in ERK activity after LBH589 treatment in prostate cancer cell lines. The cells were treated with or without 75 nM LBH589 for 24 h and analyzed by immuno-blotting. (B) DU 145 was treated with 50 nM LBH589 or combined pre-treatment with 10 µM UO126. The lysates were immuno-blotted using indicated antibodies. The down-regulation of HDAC6 and activation of ERK were induced by LBH589 treatment, but attenuated by combining LBH589 with a MEK inhibitor (UO126) treatment. (C) LBH589 induced ERK activation and HDAC6 down-regulation in a dosage-dependent manner in 22Rv1. 22Rv1 cells were treated with indicated concentrations of LBH589 for 24 h. The lysates were analyzed by immuno-blotting using indicated antibodies. (**B**–**C**) DU 45 and 22Rv1 were cultured in a serum starvation conditions for 24 h before treatment.
(TIF)
