# Supplementary figures and images for: The HDAC Inhibitor LBH589 Induces ERK-Dependent Prometaphase Arrest in Prostate Cancer via HDAC6 Inactivation and Down-Regulation
Source: PLoS One. 2013 Sep 4;8(9):e73401. doi: 10.1371/journal.pone.0073401 (PMC3762759; doi:10.1371/journal.pone.0073401)

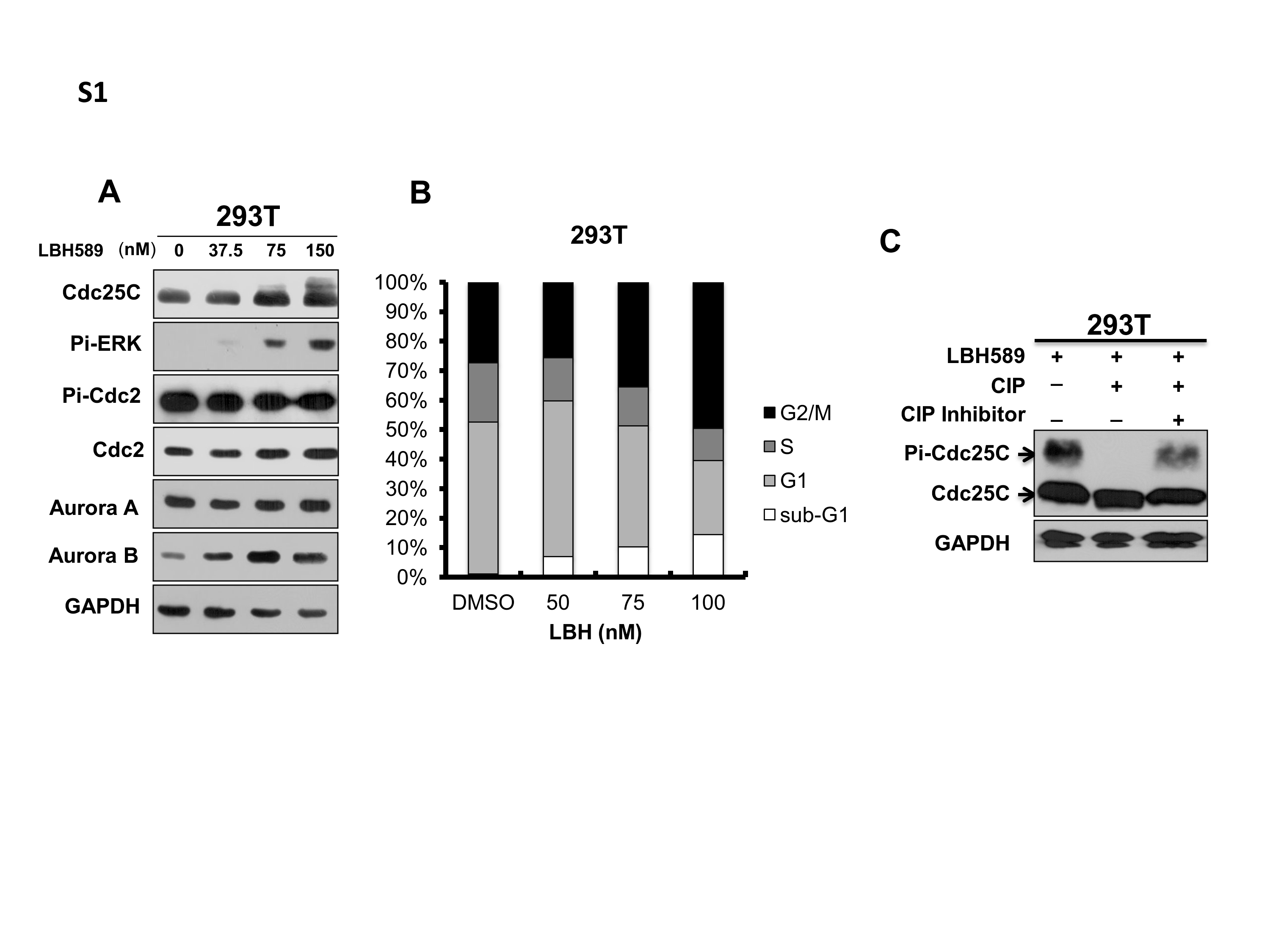

Supplement: Figure S1 — LBH589 induced ERK activation, G2/M arrest, and hyper-phosphorylation of Cdc25C in 293T. The 293T cells were treated with 37.5, 75 and 150 nM of LBH589 for 24 h. (A) The dose- and time-dependent correlation of LBH589-mediated ERK activation and Cdc25C hyper-phosphorylation. (B) The cell cycles were analyzed by PI-staining and flow cytometry according to DNA content. (C) The dephosphorylation assay. The lysates were incubated with phosphatase or combined with phosphatase inhibitor. The hyper-phosphorylated and dephosphorylated Cdc25C were analyzed by immuno-blotting with Cdc25C antibody. (TIF) [file pone.0073401.s001.tif]

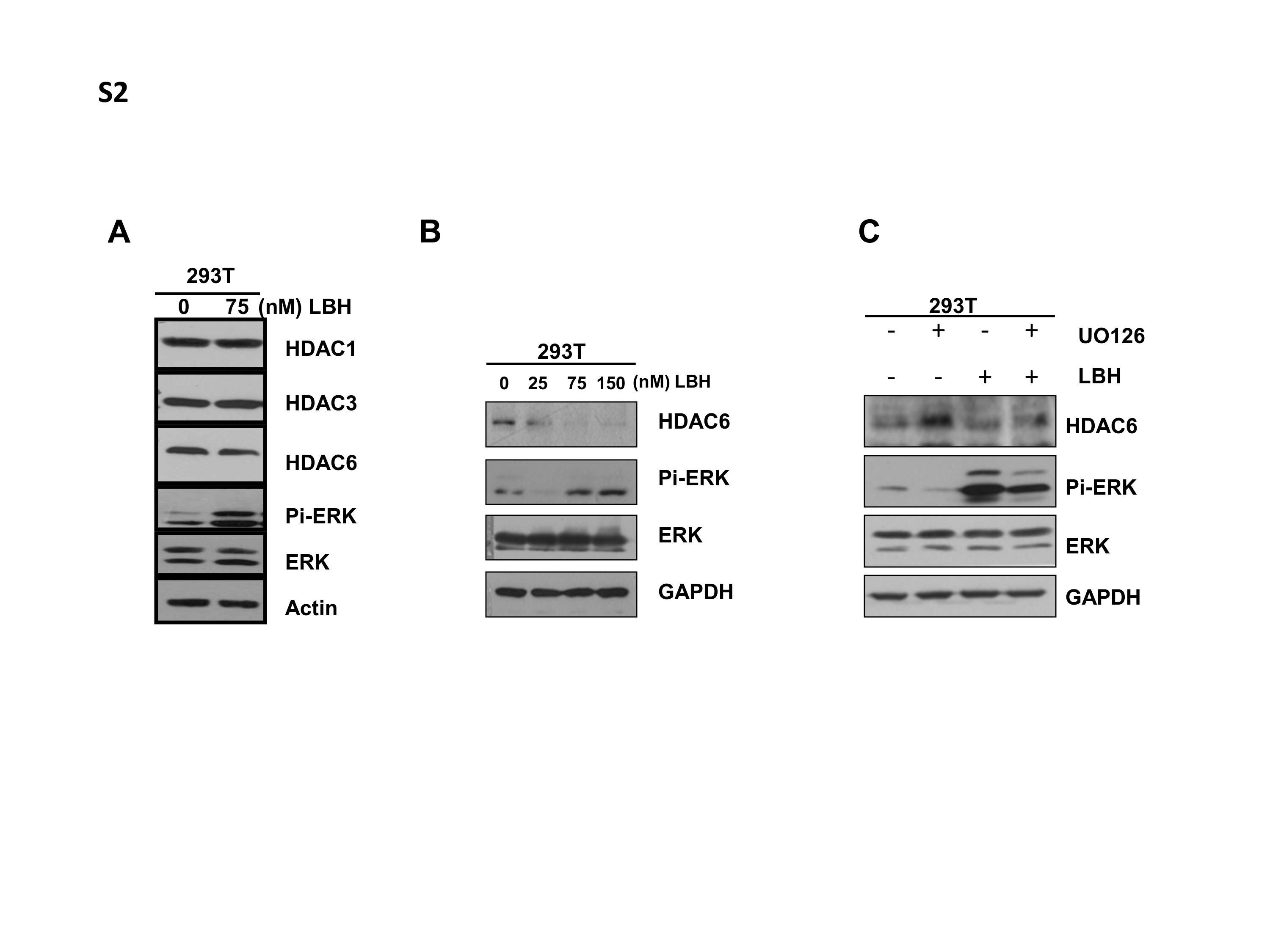

Supplement: Figure S2 — LBH589-induced down-regulation of HDAC6 correlated with ERK activation in 293T. (A) The LBH589-induced down-regulation of HDAC6 correlated with ERK activation. (B) LBH589 mediated HDAC6 down-regulation in a dose-dependent manner. (C) ERK activity was involved in the LBH589-mediated HDAC6 down-regulation, as shown by the immuno-blotting of lysates from cells treated with LBH589 or combined with UO126 pre-treatment. (TIF) [file pone.0073401.s002.tif]

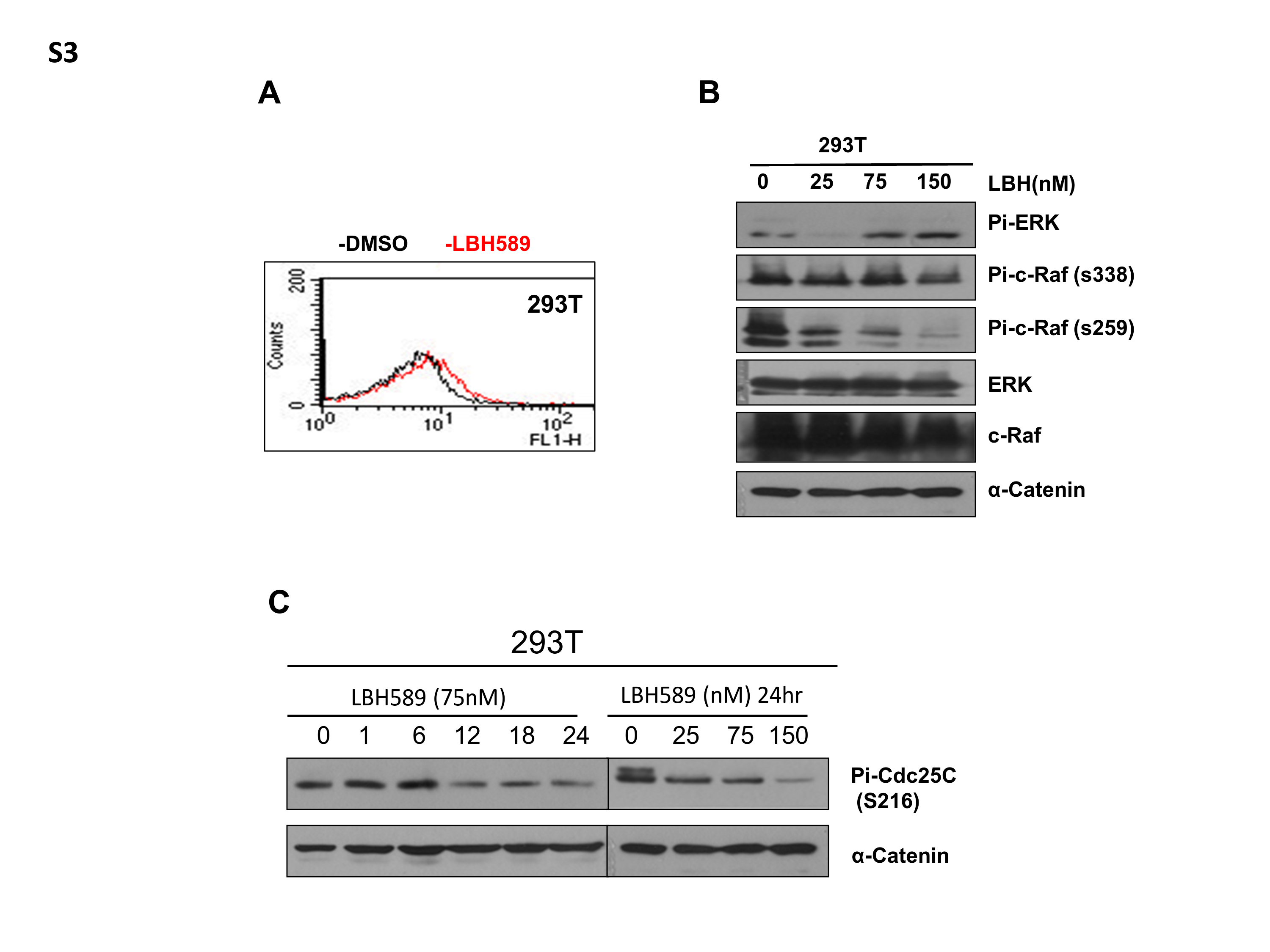

Supplement: Figure S3 — LBH589 induced ERK activation by modulating c-Raf activity. (A) Analysis of ROS production. 293T cells were treated with 75 nM LBH589 for 24 h. (B) The pattern of c-Raf signaling pathway on LBH589 treatment. 293T cells were treated with LBH589 for 24 h and the lysates were immuno-blotted with the indicated antibodies. (C) LBH589 induced the dephosphorylation of Cdc25C-Ser216. (TIF) [file pone.0073401.s003.tif]

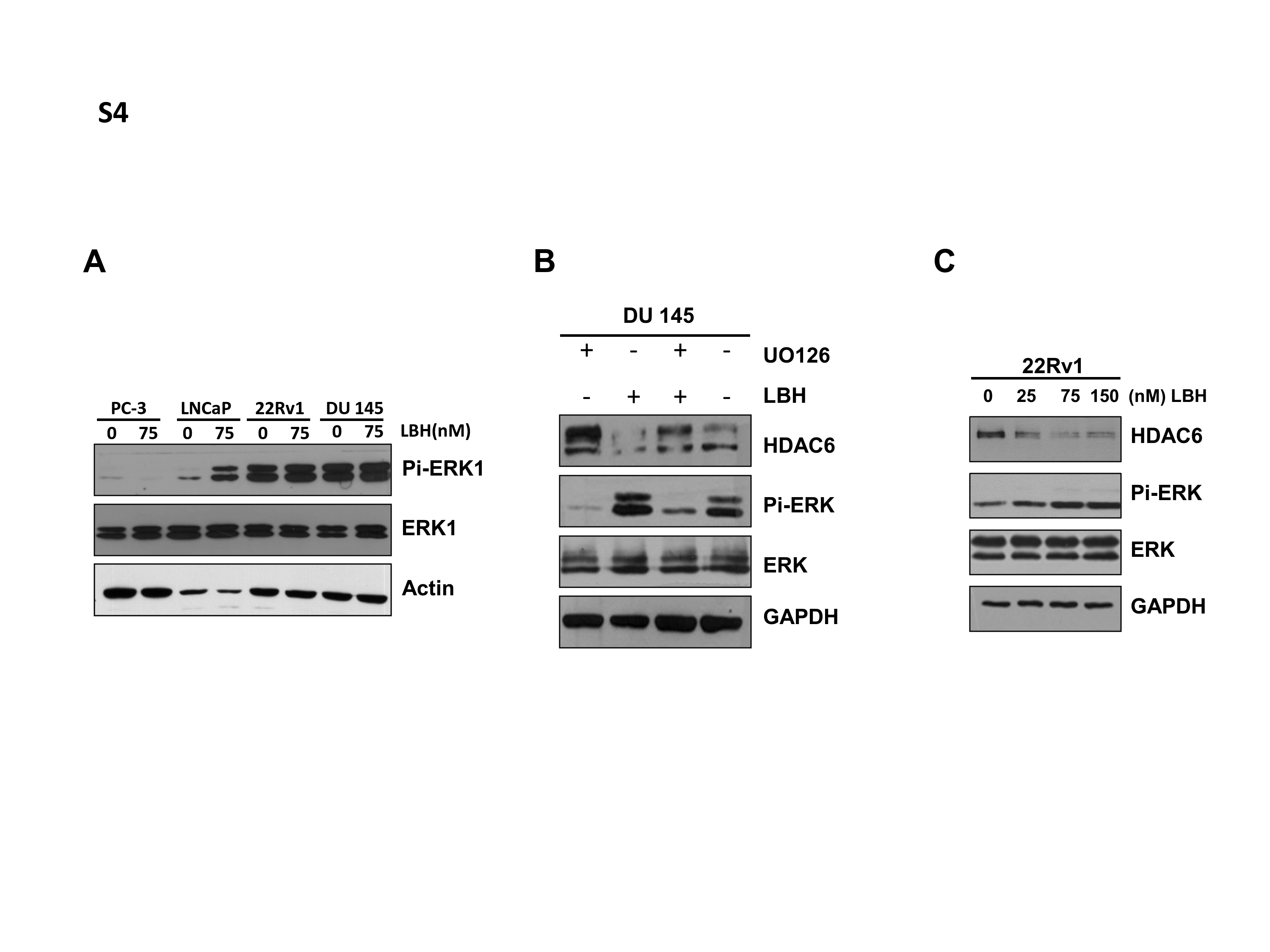

Supplement: Figure S4 — LBH589 induced ERK activation and HDAC6 down-regulation in 22Rv1 and DU 145. (A) Dynamic changes in ERK activity after LBH589 treatment in prostate cancer cell lines. The cells were treated with or without 75 nM LBH589 for 24 h and analyzed by immuno-blotting. (B) DU 145 was treated with 50 nM LBH589 or combined pre-treatment with 10 µM UO126. The lysates were immuno-blotted using indicated antibodies. The down-regulation of HDAC6 and activation of ERK were induced by LBH589 treatment, but attenuated by combining LBH589 with a MEK inhibitor (UO126) treatment. (C) LBH589 induced ERK activation and HDAC6 down-regulation in a dosage-dependent manner in 22Rv1. 22Rv1 cells were treated with indicated concentrations of LBH589 for 24 h. The lysates were analyzed by immuno-blotting using indicated antibodies. (B–C) DU 45 and 22Rv1 were cultured in a serum starvation conditions for 24 h before treatment. (TIF) [file pone.0073401.s004.tif]
